# *catena*-Poly[[triphenyl­tin(IV)]-μ-2-[(3,5-di-*tert*-butyl-4-hydroxy­benz­yl)sulfan­yl]acetato-κ^2^ *O*:*O*′]

**DOI:** 10.1107/S1600536809023150

**Published:** 2009-06-20

**Authors:** See Mun Lee, Kong Mun Lo, Hapipah Mohd Ali, Ward T. Robinson

**Affiliations:** a Department of Chemistry, University of Malaya, 50603 Kuala Lumpur, Malaysia

## Abstract

The title compound, [Sn(C_6_H_5_)_3_(C_17_H_25_O_3_S)]_*n*_, comprises two symmetry-independent five-coordinated triphenyl­tin mol­ecules which are linked by carboxyl­ate bridges into a polymeric chain. The Sn^IV^ atom is in a distorted *trans*-C_3_SnO_2_ trigonal-bipyramidal geometry. The presence of two bulky *tert*-butyl groups on the benzene ring prevents any hydrogen-bonding inter­actions involving the hydroxyl substituents.

## Related literature

For chemical background, see: Yehye *et al.* (2009 ▶). For related structures, see: Tiekink (1991 ▶); Parvez *et al.* (2002 ▶); Zhang *et al.* (2007 ▶).
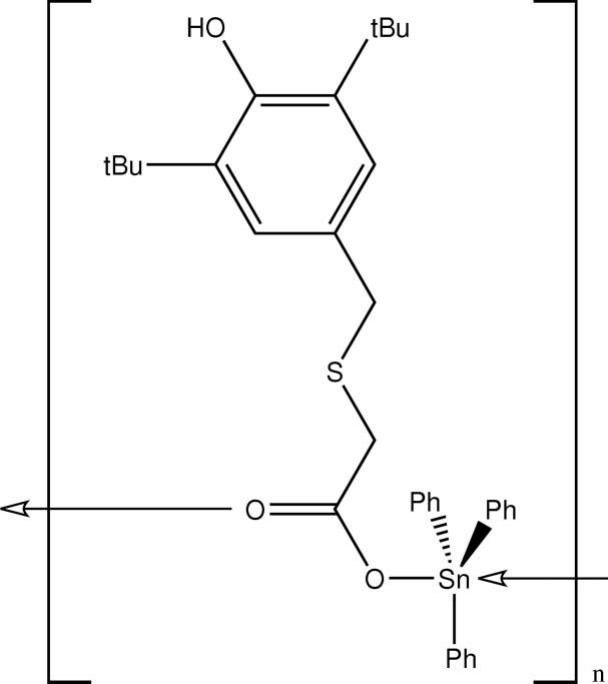


## Experimental

### 

#### Crystal data

[Sn(C_6_H_5_)_3_(C_17_H_25_O_3_S)]
*M* *_r_* = 1318.84
Monoclinic, 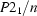

*a* = 23.1141 (3) Å
*b* = 10.6933 (1) Å
*c* = 26.4904 (3) Å
β = 105.318 (1)°
*V* = 6314.92 (12) Å^3^
*Z* = 4
Mo *K*α radiation
μ = 0.91 mm^−1^
*T* = 100 K
0.33 × 0.12 × 0.05 mm

#### Data collection

Bruker APEXII CCD area-detector diffractometer
Absorption correction: multi-scan (*SADABS*; Sheldrick, 1996 ▶) *T*_min_ = 0.754, *T*_max_ = 0.960
46753 measured reflections
14430 independent reflections
9830 reflections with *I* > 2σ(*I*)
*R*_int_ = 0.058

#### Refinement

*R*[*F* ^2^ > 2σ(*F* ^2^)] = 0.041
*wR*(*F* ^2^) = 0.094
*S* = 1.01
14430 reflections
735 parameters
H-atom parameters constrained
Δρ_max_ = 0.91 e Å^−3^
Δρ_min_ = −0.60 e Å^−3^

### 

Data collection: *APEX2* (Bruker, 2008 ▶); cell refinement: *SAINT* (Bruker, 2008 ▶); data reduction: *SAINT*; program(s) used to solve structure: *SHELXS97* (Sheldrick, 2008 ▶); program(s) used to refine structure: *SHELXL97* (Sheldrick, 2008 ▶); molecular graphics: *X-SEED* (Barbour, 2001 ▶); software used to prepare material for publication: *SHELXTL* (Sheldrick, 2008 ▶).

## Supplementary Material

Crystal structure: contains datablocks I, global. DOI: 10.1107/S1600536809023150/tk2461sup1.cif

Structure factors: contains datablocks I. DOI: 10.1107/S1600536809023150/tk2461Isup2.hkl

Additional supplementary materials:  crystallographic information; 3D view; checkCIF report

## Figures and Tables

**Fig. 1.**
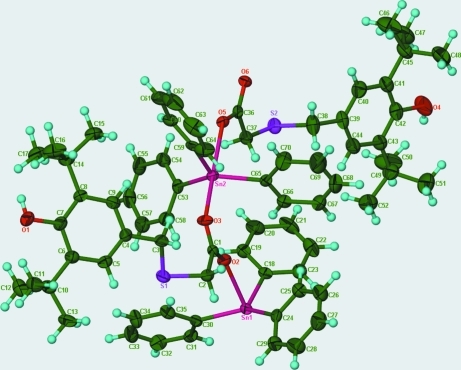
Thermal ellipsoid plot (Barbour, 2001) of the asymmetric unit in (I) drawn at the 70% probability level. Hydrogen atoms are drawn as spheres of arbitrary radius.

## supplementary crystallographic information

### Comment

Organotin carboxylates have received much attention due to their extensive applications in different fields of science, including biological activity (Yehye *et al.*, 2009). Triorganotin carboxylates are either monomeric or polymeric, depending on the steric bulk of the organic substituents. In the case of polymeric structures, the asymmetric triorganotin fragments are linked by strong carboxylate bridges (Tiekink, 1991; Parvez *et al.* 2002; Zhang *et al.*, 2007).

The structure of the title compound, (I), contains two symmetry unrelated triphenyltin units which form a polymeric chain by strong carboxylate bridges. The tin atom in the polymeric unit adopts the trigonal bipyramidal geometry in which the axial positions are occupied by the carboxylate-O atoms of the adjacent 2-(3,5-di-*tert*-4-hydroxybenzyl)sulfanylacetate ligands. The Sn—O bond distances of 2.186 (2) Å, 2.452 (2) Å and 2.151 (2) Å, 2.384 (2) Å are in good agreement with values reported for many polymeric triorganotin carboxylates (Tiekink, 1991).

### Experimental

Compound (I) was prepared by refluxing 2-(3,5-di-*tert*-4-hydroxybenzyl)sulfanylacetic acid (0.39 g, 1 mmol) with triphenyltin hydroxide (0.37 g, 1 mmol) in absolute ethanol for 2 h. Colourless crystals were obtained by slow evaporation at room temperature.

### Refinement

Hydrogen atoms were placed at calculated positions (C–H 0.95 to 0.99; O–H 0.84 Å) and were treated as riding on their parent atoms, with *U*(H) set to 1.2–1.5 times *U*_eq_(C,*N*,*O*).

### Crystal data

**Table:**

[Sn(C_6_H_5_)_3_(C_17_H_25_O_3_S)]	*F*(000) = 2720
*M**_r_* = 1318.84	*D*_x_ = 1.387 Mg m^−^^3^
Monoclinic, *P*2_1_/*n*	Mo *K*α radiation, λ = 0.71073 Å
Hall symbol: -P 2yn	Cell parameters from 7156 reflections
*a* = 23.1141 (3) Å	θ = 2.3–27.8°
*b* = 10.6933 (1) Å	µ = 0.91 mm^−^^1^
*c* = 26.4904 (3) Å	*T* = 100 K
β = 105.318 (1)°	Plate, colourless
*V* = 6314.92 (12) Å^3^	0.33 × 0.12 × 0.05 mm
*Z* = 4	

### Data collection

**Table:**

Bruker APEXII CCD area-detector diffractometer	14430 independent reflections
Radiation source: fine-focus sealed tube	9830 reflections with *I* > 2σ(*I*)
graphite	*R*_int_ = 0.058
φ and ω scans	θ_max_ = 27.5°, θ_min_ = 1.0°
Absorption correction: multi-scan (*SADABS*; Sheldrick, 1996)	*h* = −30→30
*T*_min_ = 0.754, *T*_max_ = 0.960	*k* = −13→13
46753 measured reflections	*l* = −34→34

### Refinement

**Table:**

Refinement on *F*^2^	Primary atom site location: structure-invariant direct methods
Least-squares matrix: full	Secondary atom site location: difference Fourier map
*R*[*F*^2^ > 2σ(*F*^2^)] = 0.041	Hydrogen site location: inferred from neighbouring sites
*wR*(*F*^2^) = 0.094	H-atom parameters constrained
*S* = 1.01	*w* = 1/[σ^2^(*F*_o_^2^) + (0.0347*P*)^2^ + 5.3025*P*] where *P* = (*F*_o_^2^ + 2*F*_c_^2^)/3
14430 reflections	(Δ/σ)_max_ = 0.003
735 parameters	Δρ_max_ = 0.91 e Å^−^^3^
0 restraints	Δρ_min_ = −0.60 e Å^−^^3^

### Special details

**Table:**

Geometry. All e.s.d.'s (except the e.s.d. in the dihedral angle between two l.s. planes) are estimated using the full covariance matrix. The cell e.s.d.'s are taken into account individually in the estimation of e.s.d.'s in distances, angles and torsion angles; correlations between e.s.d.'s in cell parameters are only used when they are defined by crystal symmetry. An approximate (isotropic) treatment of cell e.s.d.'s is used for estimating e.s.d.'s involving l.s. planes.
Refinement. Refinement of *F*^2^ against ALL reflections. The weighted *R*-factor *wR* and goodness of fit *S* are based on *F*^2^, conventional *R*-factors *R* are based on *F*, with *F* set to zero for negative *F*^2^. The threshold expression of *F*^2^ > σ(*F*^2^) is used only for calculating *R*-factors(gt) *etc*. and is not relevant to the choice of reflections for refinement. *R*-factors based on *F*^2^ are statistically about twice as large as those based on *F*, and *R*- factors based on ALL data will be even larger.

### Fractional atomic coordinates and isotropic or equivalent isotropic displacement parameters (Å^2^)

**Table:**

	*x*	*y*	*z*	*U*_iso_*/*U*_eq_	
Sn1	0.910774 (10)	1.12298 (2)	0.143808 (9)	0.01987 (7)	
Sn2	0.844590 (10)	0.61317 (2)	0.124459 (9)	0.01865 (6)	
O1	0.43868 (12)	0.6917 (3)	0.10995 (13)	0.0361 (7)	
H1	0.4406	0.6170	0.1200	0.054*	
O2	0.85157 (11)	0.9306 (2)	0.12252 (10)	0.0234 (6)	
O3	0.78885 (11)	0.7715 (2)	0.09407 (10)	0.0248 (6)	
O4	1.33300 (12)	0.4480 (3)	0.15895 (14)	0.0507 (9)	
H4	1.3521	0.5037	0.1789	0.076*	
O5	0.89756 (10)	0.4208 (2)	0.14556 (9)	0.0212 (5)	
O6	0.97151 (11)	0.2825 (2)	0.16497 (10)	0.0238 (6)	
S1	0.68509 (4)	0.95913 (9)	0.08727 (4)	0.0254 (2)	
S2	1.07276 (4)	0.46546 (9)	0.21041 (4)	0.0272 (2)	
C1	0.80231 (15)	0.8877 (3)	0.09626 (14)	0.0209 (7)	
C2	0.75315 (15)	0.9722 (3)	0.06523 (15)	0.0230 (8)	
H2A	0.7440	0.9497	0.0277	0.028*	
H2B	0.7673	1.0599	0.0690	0.028*	
C3	0.64945 (16)	0.8224 (3)	0.05085 (14)	0.0227 (8)	
H3A	0.6395	0.8404	0.0129	0.027*	
H3B	0.6777	0.7510	0.0581	0.027*	
C4	0.59296 (15)	0.7880 (3)	0.06610 (13)	0.0207 (8)	
C5	0.55189 (15)	0.8788 (3)	0.07229 (13)	0.0227 (8)	
H5	0.5595	0.9640	0.0662	0.027*	
C6	0.50024 (16)	0.8484 (3)	0.08713 (14)	0.0221 (8)	
C7	0.48971 (16)	0.7211 (4)	0.09447 (15)	0.0258 (8)	
C8	0.52889 (16)	0.6268 (3)	0.08767 (14)	0.0240 (8)	
C9	0.58075 (16)	0.6635 (3)	0.07387 (14)	0.0242 (8)	
H9	0.6085	0.6013	0.0697	0.029*	
C10	0.45783 (16)	0.9504 (4)	0.09774 (15)	0.0256 (8)	
C11	0.39367 (16)	0.9309 (4)	0.06322 (16)	0.0313 (9)	
H11A	0.3794	0.8478	0.0699	0.047*	
H11B	0.3935	0.9375	0.0263	0.047*	
H11C	0.3672	0.9949	0.0715	0.047*	
C12	0.45897 (17)	0.9485 (4)	0.15577 (15)	0.0330 (10)	
H12A	0.4999	0.9648	0.1770	0.049*	
H12B	0.4458	0.8663	0.1648	0.049*	
H12C	0.4319	1.0131	0.1626	0.049*	
C13	0.47751 (18)	1.0811 (4)	0.08518 (17)	0.0336 (10)	
H13A	0.4504	1.1438	0.0932	0.050*	
H13B	0.4762	1.0858	0.0480	0.050*	
H13C	0.5185	1.0971	0.1063	0.050*	
C14	0.51615 (17)	0.4867 (4)	0.09642 (16)	0.0305 (9)	
C15	0.5637 (2)	0.4028 (4)	0.08419 (19)	0.0408 (11)	
H15A	0.6031	0.4239	0.1075	0.061*	
H15B	0.5645	0.4152	0.0477	0.061*	
H15C	0.5544	0.3152	0.0895	0.061*	
C16	0.4552 (2)	0.4450 (4)	0.06023 (19)	0.0444 (12)	
H16A	0.4539	0.4649	0.0238	0.067*	
H16B	0.4227	0.4891	0.0701	0.067*	
H16C	0.4504	0.3547	0.0638	0.067*	
C17	0.5170 (2)	0.4630 (4)	0.15427 (17)	0.0391 (11)	
H17A	0.5120	0.3734	0.1596	0.059*	
H17B	0.4842	0.5094	0.1627	0.059*	
H17C	0.5554	0.4911	0.1771	0.059*	
C18	0.98565 (16)	1.0026 (3)	0.17101 (14)	0.0219 (8)	
C19	0.98579 (17)	0.9159 (3)	0.21037 (15)	0.0269 (9)	
H19	0.9524	0.9118	0.2249	0.032*	
C20	1.03377 (19)	0.8360 (4)	0.22860 (16)	0.0346 (10)	
H20	1.0333	0.7777	0.2555	0.042*	
C21	1.08251 (19)	0.8412 (4)	0.20758 (17)	0.0397 (11)	
H21	1.1153	0.7856	0.2197	0.048*	
C22	1.08343 (18)	0.9270 (4)	0.16914 (17)	0.0365 (10)	
H22	1.1171	0.9308	0.1550	0.044*	
C23	1.03558 (16)	1.0082 (4)	0.15078 (15)	0.0278 (9)	
H23	1.0368	1.0676	0.1244	0.033*	
C24	0.88567 (15)	1.1508 (3)	0.06164 (14)	0.0214 (8)	
C25	0.90572 (18)	1.0625 (4)	0.03130 (16)	0.0304 (9)	
H25	0.9292	0.9936	0.0478	0.037*	
C26	0.89162 (19)	1.0745 (4)	−0.02270 (16)	0.0352 (10)	
H26	0.9056	1.0141	−0.0430	0.042*	
C27	0.85748 (19)	1.1736 (4)	−0.04711 (17)	0.0374 (10)	
H27	0.8482	1.1820	−0.0841	0.045*	
C28	0.83697 (19)	1.2603 (4)	−0.01776 (16)	0.0357 (10)	
H28	0.8129	1.3281	−0.0346	0.043*	
C29	0.85112 (17)	1.2494 (4)	0.03653 (15)	0.0296 (9)	
H29	0.8369	1.3103	0.0565	0.035*	
C30	0.86841 (15)	1.1538 (3)	0.20472 (14)	0.0201 (8)	
C31	0.90234 (17)	1.2129 (3)	0.24995 (14)	0.0258 (8)	
H31	0.9415	1.2423	0.2513	0.031*	
C32	0.87923 (18)	1.2292 (4)	0.29295 (15)	0.0311 (9)	
H32	0.9024	1.2707	0.3233	0.037*	
C33	0.82320 (18)	1.1858 (4)	0.29177 (16)	0.0325 (10)	
H33	0.8076	1.1972	0.3212	0.039*	
C34	0.78947 (16)	1.1258 (4)	0.24807 (15)	0.0313 (9)	
H34	0.7506	1.0957	0.2474	0.038*	
C35	0.81167 (16)	1.1088 (4)	0.20505 (15)	0.0267 (8)	
H35	0.7881	1.0661	0.1752	0.032*	
C36	0.95145 (15)	0.3923 (3)	0.16535 (14)	0.0207 (8)	
C37	0.99361 (15)	0.4947 (4)	0.19353 (15)	0.0242 (8)	
H37A	0.9825	0.5149	0.2262	0.029*	
H37B	0.9858	0.5705	0.1713	0.029*	
C38	1.08468 (16)	0.4698 (4)	0.14585 (15)	0.0345 (10)	
H38A	1.0642	0.3979	0.1251	0.041*	
H38B	1.0673	0.5476	0.1278	0.041*	
C39	1.15088 (17)	0.4648 (4)	0.14936 (15)	0.0300 (9)	
C40	1.17645 (17)	0.3574 (4)	0.13547 (15)	0.0319 (9)	
H40	1.1517	0.2867	0.1236	0.038*	
C41	1.23752 (17)	0.3501 (4)	0.13841 (15)	0.0299 (9)	
C42	1.27254 (17)	0.4565 (4)	0.15695 (17)	0.0338 (10)	
C43	1.24881 (17)	0.5674 (4)	0.17203 (16)	0.0304 (9)	
C44	1.18727 (17)	0.5676 (4)	0.16737 (16)	0.0318 (9)	
H44	1.1695	0.6410	0.1769	0.038*	
C45	1.26454 (19)	0.2303 (4)	0.12188 (17)	0.0363 (10)	
C46	1.2171 (2)	0.1292 (5)	0.1035 (2)	0.0577 (14)	
H46A	1.1998	0.1066	0.1323	0.087*	
H46B	1.2355	0.0553	0.0924	0.087*	
H46C	1.1853	0.1609	0.0740	0.087*	
C47	1.3130 (2)	0.1754 (5)	0.16756 (19)	0.0512 (13)	
H47A	1.3454	0.2364	0.1793	0.077*	
H47B	1.3291	0.0989	0.1561	0.077*	
H47C	1.2955	0.1559	0.1965	0.077*	
C48	1.2906 (2)	0.2593 (5)	0.0759 (2)	0.0566 (14)	
H48A	1.2587	0.2900	0.0464	0.085*	
H48B	1.3081	0.1831	0.0656	0.085*	
H48C	1.3218	0.3233	0.0864	0.085*	
C49	1.28762 (18)	0.6834 (4)	0.19216 (17)	0.0347 (10)	
C50	1.3375 (2)	0.6528 (5)	0.2423 (2)	0.0594 (15)	
H50A	1.3582	0.7301	0.2568	0.089*	
H50B	1.3664	0.5949	0.2337	0.089*	
H50C	1.3197	0.6141	0.2682	0.089*	
C51	1.3139 (2)	0.7366 (5)	0.1493 (2)	0.0556 (14)	
H51A	1.2812	0.7621	0.1193	0.083*	
H51B	1.3383	0.6725	0.1383	0.083*	
H51C	1.3390	0.8092	0.1630	0.083*	
C52	1.2512 (2)	0.7903 (4)	0.20762 (19)	0.0437 (11)	
H52A	1.2193	0.8159	0.1770	0.065*	
H52B	1.2777	0.8616	0.2203	0.065*	
H52C	1.2335	0.7617	0.2353	0.065*	
C53	0.85574 (15)	0.6475 (3)	0.20566 (14)	0.0218 (8)	
C54	0.86807 (15)	0.5479 (4)	0.24119 (15)	0.0263 (9)	
H54	0.8732	0.4662	0.2290	0.032*	
C55	0.87288 (17)	0.5667 (4)	0.29420 (16)	0.0321 (10)	
H55	0.8817	0.4985	0.3180	0.039*	
C56	0.86483 (18)	0.6842 (4)	0.31174 (16)	0.0330 (10)	
H56	0.8690	0.6975	0.3480	0.040*	
C57	0.85087 (18)	0.7825 (4)	0.27763 (16)	0.0333 (10)	
H57	0.8443	0.8630	0.2903	0.040*	
C58	0.84617 (17)	0.7656 (4)	0.22424 (15)	0.0292 (9)	
H58	0.8365	0.8343	0.2008	0.035*	
C59	0.77265 (15)	0.4907 (3)	0.08986 (15)	0.0226 (8)	
C60	0.75968 (17)	0.3891 (3)	0.11778 (17)	0.0296 (9)	
H60	0.7799	0.3803	0.1538	0.036*	
C61	0.71789 (19)	0.3009 (4)	0.0939 (2)	0.0439 (12)	
H61	0.7094	0.2320	0.1134	0.053*	
C62	0.68839 (19)	0.3129 (5)	0.0415 (2)	0.0501 (14)	
H62	0.6603	0.2509	0.0249	0.060*	
C63	0.69929 (19)	0.4135 (5)	0.01317 (19)	0.0467 (13)	
H63	0.6784	0.4223	−0.0227	0.056*	
C64	0.74144 (17)	0.5028 (4)	0.03773 (17)	0.0353 (10)	
H64	0.7488	0.5730	0.0184	0.042*	
C65	0.90345 (16)	0.6477 (3)	0.07627 (14)	0.0213 (8)	
C66	0.94946 (17)	0.7339 (4)	0.08737 (16)	0.0287 (9)	
H66	0.9560	0.7829	0.1183	0.034*	
C67	0.98644 (19)	0.7502 (4)	0.05405 (18)	0.0374 (10)	
H67	1.0181	0.8097	0.0624	0.045*	
C68	0.9774 (2)	0.6811 (4)	0.00956 (18)	0.0438 (12)	
H68	1.0028	0.6920	−0.0131	0.053*	
C69	0.9313 (2)	0.5950 (5)	−0.00272 (18)	0.0517 (13)	
H69	0.9244	0.5476	−0.0341	0.062*	
C70	0.8953 (2)	0.5781 (4)	0.03055 (17)	0.0418 (11)	
H70	0.8641	0.5175	0.0221	0.050*	

### Atomic displacement parameters (Å^2^)

**Table:**

	*U*^11^	*U*^22^	*U*^33^	*U*^12^	*U*^13^	*U*^23^
Sn1	0.02171 (13)	0.01705 (13)	0.02198 (13)	0.00369 (10)	0.00778 (10)	0.00160 (10)
Sn2	0.01963 (12)	0.01487 (12)	0.02168 (13)	−0.00021 (10)	0.00586 (9)	0.00112 (10)
O1	0.0296 (15)	0.0267 (16)	0.059 (2)	0.0013 (12)	0.0243 (14)	0.0093 (14)
O2	0.0244 (14)	0.0190 (13)	0.0269 (15)	−0.0012 (11)	0.0069 (11)	0.0014 (11)
O3	0.0237 (13)	0.0138 (13)	0.0354 (16)	−0.0001 (10)	0.0049 (11)	−0.0008 (11)
O4	0.0245 (16)	0.052 (2)	0.081 (3)	−0.0008 (15)	0.0221 (16)	−0.0155 (18)
O5	0.0225 (13)	0.0173 (13)	0.0250 (14)	0.0013 (10)	0.0084 (11)	0.0029 (10)
O6	0.0231 (13)	0.0176 (13)	0.0329 (15)	0.0035 (11)	0.0114 (11)	0.0003 (11)
S1	0.0238 (5)	0.0209 (5)	0.0328 (6)	−0.0008 (4)	0.0096 (4)	−0.0053 (4)
S2	0.0194 (5)	0.0323 (6)	0.0296 (5)	0.0014 (4)	0.0062 (4)	0.0005 (4)
C1	0.0237 (18)	0.0189 (18)	0.0231 (19)	0.0033 (16)	0.0116 (15)	0.0008 (16)
C2	0.0216 (18)	0.0185 (19)	0.030 (2)	0.0000 (15)	0.0090 (16)	0.0005 (16)
C3	0.0251 (19)	0.0186 (19)	0.024 (2)	−0.0011 (15)	0.0062 (15)	−0.0049 (15)
C4	0.0235 (18)	0.0217 (19)	0.0168 (18)	−0.0011 (15)	0.0051 (14)	0.0002 (14)
C5	0.0268 (19)	0.0188 (18)	0.0209 (18)	−0.0011 (16)	0.0035 (14)	0.0025 (15)
C6	0.0216 (18)	0.0212 (19)	0.0218 (19)	0.0043 (15)	0.0026 (15)	0.0036 (15)
C7	0.0220 (19)	0.024 (2)	0.032 (2)	−0.0014 (16)	0.0090 (16)	0.0035 (17)
C8	0.0276 (19)	0.0189 (19)	0.027 (2)	−0.0001 (16)	0.0091 (15)	0.0027 (16)
C9	0.028 (2)	0.0216 (19)	0.023 (2)	0.0036 (16)	0.0063 (16)	−0.0002 (15)
C10	0.0211 (19)	0.023 (2)	0.031 (2)	0.0038 (15)	0.0045 (16)	−0.0017 (16)
C11	0.025 (2)	0.029 (2)	0.038 (2)	0.0053 (17)	0.0044 (17)	0.0006 (18)
C12	0.028 (2)	0.041 (3)	0.031 (2)	0.0006 (18)	0.0106 (18)	−0.0063 (19)
C13	0.033 (2)	0.021 (2)	0.045 (3)	0.0070 (17)	0.0080 (19)	−0.0033 (18)
C14	0.033 (2)	0.021 (2)	0.038 (2)	0.0013 (17)	0.0098 (18)	0.0037 (17)
C15	0.051 (3)	0.020 (2)	0.057 (3)	0.0020 (19)	0.024 (2)	0.005 (2)
C16	0.046 (3)	0.024 (2)	0.056 (3)	−0.005 (2)	0.000 (2)	0.000 (2)
C17	0.044 (3)	0.027 (2)	0.048 (3)	0.0013 (19)	0.015 (2)	0.008 (2)
C18	0.0234 (19)	0.0185 (18)	0.023 (2)	0.0012 (15)	0.0052 (15)	−0.0038 (15)
C19	0.029 (2)	0.025 (2)	0.027 (2)	0.0020 (16)	0.0097 (17)	−0.0002 (16)
C20	0.046 (3)	0.030 (2)	0.025 (2)	0.0119 (19)	0.0031 (19)	0.0026 (17)
C21	0.036 (2)	0.042 (3)	0.037 (3)	0.020 (2)	0.003 (2)	−0.008 (2)
C22	0.024 (2)	0.048 (3)	0.038 (3)	0.0068 (19)	0.0082 (18)	−0.009 (2)
C23	0.027 (2)	0.028 (2)	0.029 (2)	−0.0023 (17)	0.0084 (16)	−0.0023 (17)
C24	0.0211 (18)	0.0227 (19)	0.0224 (19)	−0.0035 (15)	0.0093 (15)	−0.0002 (15)
C25	0.034 (2)	0.027 (2)	0.033 (2)	0.0020 (17)	0.0140 (18)	−0.0005 (17)
C26	0.043 (2)	0.035 (2)	0.031 (2)	0.000 (2)	0.017 (2)	−0.0073 (19)
C27	0.043 (3)	0.046 (3)	0.024 (2)	−0.007 (2)	0.0098 (19)	−0.0023 (19)
C28	0.041 (2)	0.035 (2)	0.028 (2)	0.0103 (19)	0.0031 (18)	0.0034 (19)
C29	0.036 (2)	0.028 (2)	0.026 (2)	0.0018 (18)	0.0123 (17)	−0.0006 (17)
C30	0.0221 (18)	0.0184 (18)	0.0203 (19)	0.0042 (14)	0.0062 (14)	0.0010 (14)
C31	0.030 (2)	0.022 (2)	0.026 (2)	−0.0007 (16)	0.0086 (16)	0.0038 (16)
C32	0.039 (2)	0.031 (2)	0.021 (2)	−0.0049 (18)	0.0030 (17)	−0.0035 (17)
C33	0.037 (2)	0.039 (3)	0.025 (2)	0.0041 (19)	0.0135 (18)	0.0001 (18)
C34	0.0233 (19)	0.043 (3)	0.030 (2)	0.0026 (19)	0.0118 (16)	−0.0023 (19)
C35	0.028 (2)	0.026 (2)	0.025 (2)	0.0037 (17)	0.0054 (16)	−0.0042 (17)
C36	0.0204 (17)	0.024 (2)	0.0206 (18)	0.0048 (15)	0.0101 (14)	0.0055 (15)
C37	0.0190 (18)	0.025 (2)	0.029 (2)	0.0006 (15)	0.0069 (15)	−0.0011 (16)
C38	0.022 (2)	0.053 (3)	0.029 (2)	−0.0011 (19)	0.0082 (17)	−0.002 (2)
C39	0.024 (2)	0.042 (3)	0.026 (2)	0.0005 (18)	0.0099 (16)	−0.0040 (18)
C40	0.029 (2)	0.036 (2)	0.030 (2)	−0.0060 (18)	0.0075 (17)	−0.0019 (18)
C41	0.031 (2)	0.033 (2)	0.026 (2)	0.0037 (17)	0.0098 (17)	−0.0025 (17)
C42	0.023 (2)	0.043 (3)	0.038 (3)	0.0008 (18)	0.0123 (18)	−0.001 (2)
C43	0.027 (2)	0.030 (2)	0.036 (2)	0.0010 (17)	0.0118 (18)	−0.0007 (18)
C44	0.027 (2)	0.033 (2)	0.038 (2)	−0.0001 (17)	0.0125 (18)	−0.0041 (19)
C45	0.042 (2)	0.036 (2)	0.034 (2)	0.011 (2)	0.0147 (19)	−0.0034 (19)
C46	0.065 (3)	0.042 (3)	0.064 (4)	0.006 (3)	0.012 (3)	−0.019 (3)
C47	0.060 (3)	0.047 (3)	0.044 (3)	0.023 (2)	0.009 (2)	0.000 (2)
C48	0.082 (4)	0.046 (3)	0.053 (3)	0.023 (3)	0.038 (3)	0.004 (3)
C49	0.028 (2)	0.032 (2)	0.043 (3)	−0.0039 (18)	0.0092 (19)	−0.0010 (19)
C50	0.053 (3)	0.041 (3)	0.069 (4)	0.002 (2)	−0.010 (3)	−0.011 (3)
C51	0.047 (3)	0.054 (3)	0.075 (4)	−0.006 (3)	0.032 (3)	0.006 (3)
C52	0.046 (3)	0.035 (3)	0.051 (3)	−0.003 (2)	0.014 (2)	−0.003 (2)
C53	0.0162 (17)	0.027 (2)	0.024 (2)	−0.0027 (15)	0.0077 (14)	−0.0017 (15)
C54	0.0212 (19)	0.028 (2)	0.030 (2)	0.0053 (16)	0.0062 (16)	0.0013 (17)
C55	0.029 (2)	0.043 (3)	0.026 (2)	0.0063 (19)	0.0107 (17)	0.0104 (19)
C56	0.032 (2)	0.047 (3)	0.022 (2)	−0.005 (2)	0.0116 (17)	−0.0035 (19)
C57	0.036 (2)	0.033 (2)	0.033 (2)	−0.0068 (19)	0.0141 (18)	−0.0088 (19)
C58	0.032 (2)	0.026 (2)	0.029 (2)	−0.0043 (17)	0.0073 (17)	−0.0037 (17)
C59	0.0195 (18)	0.0143 (18)	0.034 (2)	0.0053 (14)	0.0064 (15)	−0.0015 (15)
C60	0.030 (2)	0.019 (2)	0.043 (2)	0.0006 (17)	0.0153 (18)	−0.0036 (18)
C61	0.035 (2)	0.024 (2)	0.079 (4)	−0.0054 (19)	0.025 (2)	−0.012 (2)
C62	0.026 (2)	0.040 (3)	0.083 (4)	−0.008 (2)	0.013 (2)	−0.033 (3)
C63	0.028 (2)	0.055 (3)	0.047 (3)	0.012 (2)	−0.010 (2)	−0.018 (2)
C64	0.030 (2)	0.028 (2)	0.042 (3)	0.0047 (18)	−0.0007 (18)	0.0014 (19)
C65	0.0267 (19)	0.0184 (19)	0.0204 (19)	0.0008 (15)	0.0093 (15)	0.0024 (14)
C66	0.035 (2)	0.022 (2)	0.032 (2)	−0.0001 (17)	0.0136 (18)	−0.0034 (17)
C67	0.038 (2)	0.030 (2)	0.052 (3)	−0.0051 (19)	0.025 (2)	0.000 (2)
C68	0.054 (3)	0.047 (3)	0.041 (3)	−0.001 (2)	0.031 (2)	0.002 (2)
C69	0.076 (4)	0.056 (3)	0.031 (3)	−0.013 (3)	0.028 (2)	−0.014 (2)
C70	0.052 (3)	0.043 (3)	0.034 (3)	−0.019 (2)	0.018 (2)	−0.012 (2)

### Geometric parameters (Å, °)

**Table:**

Sn1—C24	2.120 (4)	C30—C31	1.398 (5)
Sn1—C30	2.122 (4)	C30—C35	1.399 (5)
Sn1—C18	2.124 (3)	C31—C32	1.391 (5)
Sn1—O6^i^	2.186 (2)	C31—H31	0.9500
Sn1—O2	2.452 (2)	C32—C33	1.369 (5)
Sn2—C59	2.125 (4)	C32—H32	0.9500
Sn2—C65	2.129 (4)	C33—C34	1.372 (5)
Sn2—C53	2.130 (4)	C33—H33	0.9500
Sn2—O3	2.151 (2)	C34—C35	1.380 (5)
Sn2—O5	2.384 (2)	C34—H34	0.9500
O1—C7	1.384 (4)	C35—H35	0.9500
O1—H1	0.8400	C36—C37	1.524 (5)
O2—C1	1.254 (4)	C37—H37A	0.9900
O3—C1	1.279 (4)	C37—H37B	0.9900
O4—C42	1.387 (4)	C38—C39	1.509 (5)
O4—H4	0.8400	C38—H38A	0.9900
O5—C36	1.254 (4)	C38—H38B	0.9900
O6—C36	1.264 (4)	C39—C40	1.385 (6)
O6—Sn1^ii^	2.186 (2)	C39—C44	1.390 (5)
S1—C2	1.822 (4)	C40—C41	1.395 (5)
S1—C3	1.824 (4)	C40—H40	0.9500
S2—C37	1.792 (3)	C41—C42	1.407 (6)
S2—C38	1.803 (4)	C41—C45	1.538 (5)
C1—C2	1.513 (5)	C42—C43	1.408 (6)
C2—H2A	0.9900	C43—C44	1.395 (5)
C2—H2B	0.9900	C43—C49	1.542 (6)
C3—C4	1.511 (5)	C44—H44	0.9500
C3—H3A	0.9900	C45—C48	1.526 (6)
C3—H3B	0.9900	C45—C46	1.526 (6)
C4—C9	1.388 (5)	C45—C47	1.532 (6)
C4—C5	1.397 (5)	C46—H46A	0.9800
C5—C6	1.391 (5)	C46—H46B	0.9800
C5—H5	0.9500	C46—H46C	0.9800
C6—C7	1.405 (5)	C47—H47A	0.9800
C6—C10	1.541 (5)	C47—H47B	0.9800
C7—C8	1.399 (5)	C47—H47C	0.9800
C8—C9	1.399 (5)	C48—H48A	0.9800
C8—C14	1.555 (5)	C48—H48B	0.9800
C9—H9	0.9500	C48—H48C	0.9800
C10—C12	1.531 (5)	C49—C51	1.531 (6)
C10—C13	1.533 (5)	C49—C52	1.538 (6)
C10—C11	1.537 (5)	C49—C50	1.547 (6)
C11—H11A	0.9800	C50—H50A	0.9800
C11—H11B	0.9800	C50—H50B	0.9800
C11—H11C	0.9800	C50—H50C	0.9800
C12—H12A	0.9800	C51—H51A	0.9800
C12—H12B	0.9800	C51—H51B	0.9800
C12—H12C	0.9800	C51—H51C	0.9800
C13—H13A	0.9800	C52—H52A	0.9800
C13—H13B	0.9800	C52—H52B	0.9800
C13—H13C	0.9800	C52—H52C	0.9800
C14—C15	1.519 (6)	C53—C58	1.394 (5)
C14—C16	1.546 (5)	C53—C54	1.400 (5)
C14—C17	1.548 (6)	C54—C55	1.394 (5)
C15—H15A	0.9800	C54—H54	0.9500
C15—H15B	0.9800	C55—C56	1.369 (6)
C15—H15C	0.9800	C55—H55	0.9500
C16—H16A	0.9800	C56—C57	1.368 (6)
C16—H16B	0.9800	C56—H56	0.9500
C16—H16C	0.9800	C57—C58	1.401 (5)
C17—H17A	0.9800	C57—H57	0.9500
C17—H17B	0.9800	C58—H58	0.9500
C17—H17C	0.9800	C59—C64	1.385 (5)
C18—C19	1.394 (5)	C59—C60	1.391 (5)
C18—C23	1.396 (5)	C60—C61	1.379 (5)
C19—C20	1.382 (5)	C60—H60	0.9500
C19—H19	0.9500	C61—C62	1.381 (7)
C20—C21	1.383 (6)	C61—H61	0.9500
C20—H20	0.9500	C62—C63	1.372 (7)
C21—C22	1.375 (6)	C62—H62	0.9500
C21—H21	0.9500	C63—C64	1.396 (6)
C22—C23	1.389 (5)	C63—H63	0.9500
C22—H22	0.9500	C64—H64	0.9500
C23—H23	0.9500	C65—C66	1.379 (5)
C24—C29	1.382 (5)	C65—C70	1.391 (5)
C24—C25	1.396 (5)	C66—C67	1.392 (6)
C25—C26	1.387 (6)	C66—H66	0.9500
C25—H25	0.9500	C67—C68	1.359 (6)
C26—C27	1.376 (6)	C67—H67	0.9500
C26—H26	0.9500	C68—C69	1.380 (6)
C27—C28	1.372 (6)	C68—H68	0.9500
C27—H27	0.9500	C69—C70	1.374 (6)
C28—C29	1.393 (5)	C69—H69	0.9500
C28—H28	0.9500	C70—H70	0.9500
C29—H29	0.9500		
			
C24—Sn1—C30	134.63 (13)	C32—C31—H31	119.7
C24—Sn1—C18	114.26 (14)	C30—C31—H31	119.7
C30—Sn1—C18	109.53 (14)	C33—C32—C31	120.3 (4)
C24—Sn1—O6^i^	97.85 (12)	C33—C32—H32	119.8
C30—Sn1—O6^i^	94.48 (12)	C31—C32—H32	119.8
C18—Sn1—O6^i^	88.59 (12)	C32—C33—C34	120.1 (4)
C24—Sn1—O2	84.09 (11)	C32—C33—H33	120.0
C30—Sn1—O2	87.90 (11)	C34—C33—H33	120.0
C18—Sn1—O2	85.69 (11)	C33—C34—C35	120.4 (4)
O6^i^—Sn1—O2	174.26 (8)	C33—C34—H34	119.8
C59—Sn2—C65	113.54 (14)	C35—C34—H34	119.8
C59—Sn2—C53	114.45 (14)	C34—C35—C30	120.9 (3)
C65—Sn2—C53	130.52 (13)	C34—C35—H35	119.5
C59—Sn2—O3	90.04 (11)	C30—C35—H35	119.5
C65—Sn2—O3	93.07 (12)	O5—C36—O6	123.2 (3)
C53—Sn2—O3	98.22 (12)	O5—C36—C37	117.8 (3)
C59—Sn2—O5	82.19 (11)	O6—C36—C37	118.9 (3)
C65—Sn2—O5	85.43 (11)	C36—C37—S2	118.4 (3)
C53—Sn2—O5	89.67 (11)	C36—C37—H37A	107.7
O3—Sn2—O5	170.70 (9)	S2—C37—H37A	107.7
C7—O1—H1	109.5	C36—C37—H37B	107.7
C1—O2—Sn1	143.5 (2)	S2—C37—H37B	107.7
C1—O3—Sn2	129.3 (2)	H37A—C37—H37B	107.1
C42—O4—H4	109.5	C39—C38—S2	110.3 (3)
C36—O5—Sn2	134.3 (2)	C39—C38—H38A	109.6
C36—O6—Sn1^ii^	121.0 (2)	S2—C38—H38A	109.6
C2—S1—C3	101.51 (17)	C39—C38—H38B	109.6
C37—S2—C38	99.24 (18)	S2—C38—H38B	109.6
O2—C1—O3	123.7 (3)	H38A—C38—H38B	108.1
O2—C1—C2	121.6 (3)	C40—C39—C44	119.0 (4)
O3—C1—C2	114.6 (3)	C40—C39—C38	120.7 (4)
C1—C2—S1	111.6 (3)	C44—C39—C38	120.3 (4)
C1—C2—H2A	109.3	C39—C40—C41	121.9 (4)
S1—C2—H2A	109.3	C39—C40—H40	119.1
C1—C2—H2B	109.3	C41—C40—H40	119.1
S1—C2—H2B	109.3	C40—C41—C42	117.0 (4)
H2A—C2—H2B	108.0	C40—C41—C45	120.8 (4)
C4—C3—S1	110.8 (2)	C42—C41—C45	122.2 (4)
C4—C3—H3A	109.5	O4—C42—C41	116.2 (4)
S1—C3—H3A	109.5	O4—C42—C43	120.5 (4)
C4—C3—H3B	109.5	C41—C42—C43	123.3 (4)
S1—C3—H3B	109.5	C44—C43—C42	116.2 (4)
H3A—C3—H3B	108.1	C44—C43—C49	121.0 (4)
C9—C4—C5	118.6 (3)	C42—C43—C49	122.8 (3)
C9—C4—C3	119.8 (3)	C39—C44—C43	122.6 (4)
C5—C4—C3	121.6 (3)	C39—C44—H44	118.7
C6—C5—C4	122.1 (3)	C43—C44—H44	118.7
C6—C5—H5	118.9	C48—C45—C46	107.0 (4)
C4—C5—H5	118.9	C48—C45—C47	110.3 (4)
C5—C6—C7	117.3 (3)	C46—C45—C47	107.2 (4)
C5—C6—C10	121.4 (3)	C48—C45—C41	109.5 (4)
C7—C6—C10	121.2 (3)	C46—C45—C41	111.6 (4)
O1—C7—C8	120.4 (3)	C47—C45—C41	111.2 (4)
O1—C7—C6	117.0 (3)	C45—C46—H46A	109.5
C8—C7—C6	122.6 (3)	C45—C46—H46B	109.5
C9—C8—C7	117.4 (3)	H46A—C46—H46B	109.5
C9—C8—C14	121.2 (3)	C45—C46—H46C	109.5
C7—C8—C14	121.4 (3)	H46A—C46—H46C	109.5
C4—C9—C8	122.0 (3)	H46B—C46—H46C	109.5
C4—C9—H9	119.0	C45—C47—H47A	109.5
C8—C9—H9	119.0	C45—C47—H47B	109.5
C12—C10—C13	107.6 (3)	H47A—C47—H47B	109.5
C12—C10—C11	110.7 (3)	C45—C47—H47C	109.5
C13—C10—C11	106.8 (3)	H47A—C47—H47C	109.5
C12—C10—C6	109.3 (3)	H47B—C47—H47C	109.5
C13—C10—C6	111.5 (3)	C45—C48—H48A	109.5
C11—C10—C6	110.8 (3)	C45—C48—H48B	109.5
C10—C11—H11A	109.5	H48A—C48—H48B	109.5
C10—C11—H11B	109.5	C45—C48—H48C	109.5
H11A—C11—H11B	109.5	H48A—C48—H48C	109.5
C10—C11—H11C	109.5	H48B—C48—H48C	109.5
H11A—C11—H11C	109.5	C51—C49—C52	105.9 (4)
H11B—C11—H11C	109.5	C51—C49—C43	110.6 (4)
C10—C12—H12A	109.5	C52—C49—C43	112.4 (3)
C10—C12—H12B	109.5	C51—C49—C50	111.3 (4)
H12A—C12—H12B	109.5	C52—C49—C50	105.4 (4)
C10—C12—H12C	109.5	C43—C49—C50	111.1 (4)
H12A—C12—H12C	109.5	C49—C50—H50A	109.5
H12B—C12—H12C	109.5	C49—C50—H50B	109.5
C10—C13—H13A	109.5	H50A—C50—H50B	109.5
C10—C13—H13B	109.5	C49—C50—H50C	109.5
H13A—C13—H13B	109.5	H50A—C50—H50C	109.5
C10—C13—H13C	109.5	H50B—C50—H50C	109.5
H13A—C13—H13C	109.5	C49—C51—H51A	109.5
H13B—C13—H13C	109.5	C49—C51—H51B	109.5
C15—C14—C16	106.9 (4)	H51A—C51—H51B	109.5
C15—C14—C17	107.1 (3)	C49—C51—H51C	109.5
C16—C14—C17	109.7 (4)	H51A—C51—H51C	109.5
C15—C14—C8	111.1 (3)	H51B—C51—H51C	109.5
C16—C14—C8	111.3 (3)	C49—C52—H52A	109.5
C17—C14—C8	110.6 (3)	C49—C52—H52B	109.5
C14—C15—H15A	109.5	H52A—C52—H52B	109.5
C14—C15—H15B	109.5	C49—C52—H52C	109.5
H15A—C15—H15B	109.5	H52A—C52—H52C	109.5
C14—C15—H15C	109.5	H52B—C52—H52C	109.5
H15A—C15—H15C	109.5	C58—C53—C54	118.4 (3)
H15B—C15—H15C	109.5	C58—C53—Sn2	121.6 (3)
C14—C16—H16A	109.5	C54—C53—Sn2	119.8 (3)
C14—C16—H16B	109.5	C55—C54—C53	121.0 (4)
H16A—C16—H16B	109.5	C55—C54—H54	119.5
C14—C16—H16C	109.5	C53—C54—H54	119.5
H16A—C16—H16C	109.5	C56—C55—C54	119.5 (4)
H16B—C16—H16C	109.5	C56—C55—H55	120.2
C14—C17—H17A	109.5	C54—C55—H55	120.2
C14—C17—H17B	109.5	C57—C56—C55	120.7 (4)
H17A—C17—H17B	109.5	C57—C56—H56	119.6
C14—C17—H17C	109.5	C55—C56—H56	119.6
H17A—C17—H17C	109.5	C56—C57—C58	120.6 (4)
H17B—C17—H17C	109.5	C56—C57—H57	119.7
C19—C18—C23	118.5 (3)	C58—C57—H57	119.7
C19—C18—Sn1	119.8 (3)	C53—C58—C57	119.7 (4)
C23—C18—Sn1	121.7 (3)	C53—C58—H58	120.1
C20—C19—C18	121.0 (4)	C57—C58—H58	120.1
C20—C19—H19	119.5	C64—C59—C60	118.3 (4)
C18—C19—H19	119.5	C64—C59—Sn2	121.0 (3)
C19—C20—C21	119.9 (4)	C60—C59—Sn2	120.4 (3)
C19—C20—H20	120.1	C61—C60—C59	120.9 (4)
C21—C20—H20	120.1	C61—C60—H60	119.6
C22—C21—C20	119.9 (4)	C59—C60—H60	119.6
C22—C21—H21	120.1	C60—C61—C62	119.9 (5)
C20—C21—H21	120.1	C60—C61—H61	120.1
C21—C22—C23	120.6 (4)	C62—C61—H61	120.1
C21—C22—H22	119.7	C63—C62—C61	120.6 (4)
C23—C22—H22	119.7	C63—C62—H62	119.7
C22—C23—C18	120.1 (4)	C61—C62—H62	119.7
C22—C23—H23	120.0	C62—C63—C64	119.2 (4)
C18—C23—H23	120.0	C62—C63—H63	120.4
C29—C24—C25	118.4 (3)	C64—C63—H63	120.4
C29—C24—Sn1	124.6 (3)	C59—C64—C63	121.1 (4)
C25—C24—Sn1	117.0 (3)	C59—C64—H64	119.4
C26—C25—C24	120.5 (4)	C63—C64—H64	119.4
C26—C25—H25	119.7	C66—C65—C70	117.6 (4)
C24—C25—H25	119.7	C66—C65—Sn2	124.5 (3)
C27—C26—C25	120.4 (4)	C70—C65—Sn2	117.9 (3)
C27—C26—H26	119.8	C65—C66—C67	121.1 (4)
C25—C26—H26	119.8	C65—C66—H66	119.4
C28—C27—C26	119.6 (4)	C67—C66—H66	119.4
C28—C27—H27	120.2	C68—C67—C66	120.1 (4)
C26—C27—H27	120.2	C68—C67—H67	119.9
C27—C28—C29	120.5 (4)	C66—C67—H67	119.9
C27—C28—H28	119.8	C67—C68—C69	120.0 (4)
C29—C28—H28	119.8	C67—C68—H68	120.0
C24—C29—C28	120.6 (4)	C69—C68—H68	120.0
C24—C29—H29	119.7	C70—C69—C68	119.8 (4)
C28—C29—H29	119.7	C70—C69—H69	120.1
C31—C30—C35	117.7 (3)	C68—C69—H69	120.1
C31—C30—Sn1	117.0 (3)	C69—C70—C65	121.5 (4)
C35—C30—Sn1	125.0 (3)	C69—C70—H70	119.3
C32—C31—C30	120.6 (4)	C65—C70—H70	119.3

Symmetry codes: (i) *x*, *y*+1, *z*; (ii) *x*, *y*−1, *z*.
